# Platinum-based adjuvant chemoradiotherapy versus adjuvant radiotherapy in patients with head and neck adenoid cystic carcinoma

**DOI:** 10.1007/s00432-024-05719-0

**Published:** 2024-04-16

**Authors:** Zichen Qiu, Zheng Wu, Xiong Zhou, Minchuan Lin, Yong Su, Yalan Tao

**Affiliations:** 1 Department of Radiation Oncology, State Key Laboratory of Oncology in South China, Guangdong Provincial Clinical Research Center for Cancer, Sun Yat-Sen University Cancer Center, 651 Dongfeng Road East, Yuexiu District, Guanzhou, 510060 People’s Republic of China; 2 Department of Radiation Oncology, Hunan Cancer Hospital and The Affiliated Cancer Hospital of Xiangya School of Medicine, Central South University, 283 Tong Zi Po Road, Changsha, 410013 People’s Republic of China

**Keywords:** Adjuvant chemoradiotherapy, Postoperative radiotherapy, Head and neck adenoid cystic carcinoma, Intensity-modulated radiotherapy

## Abstract

**Purpose:**

The objective of the study was to assess the effectiveness and toxicity of platinum-based adjuvant chemoradiotherapy (POCRT) in comparison to postoperative radiotherapy (PORT) in patients with head and neck adenoid cystic carcinoma (HNACC).

**Materials and methods:**

This retrospective study analyzed patients diagnosed with HNACC at our center between January 2010 and April 2020. A 1:1 propensity score matching method was used to create a matched cohort.

**Results:**

In this study, 206 patients were analyzed, with 147 patients (71.4%) receiving postoperative radiotherapy (PORT) and 59 patients (28.6%) receiving POCRT. Twenty-one patients experienced local–regional failure. The 3-, 5-, and 10-yr local–regional control (LRC) rate for the cohort were 92.0%, 90.6%, and 86.9%, respectively. In both the entire cohort and the matched cohort, the POCRT group exhibited superior LRC compared to the PORT group (Gray's test, all P < 0.05*). Multivariate analysis identified adjuvant concurrent chemotherapy as an independent prognostic factor for LRC (Competing risks regression, HR = 0.144, 95% CI 0.026–0.802, P = 0.027*). In addition, the POCRT group had higher incidences of upper gastrointestinal toxicity and hematologic toxicities, including leukopenia, neutropenia, and anemia (all P < 0.05*).

**Conclusion:**

In terms of reducing locoregional failures in HNACC patients, POCRT may potentially offer a more effective therapeutic approach than using PORT alone, although it also entails an augmented burden of treatment-related toxicity.

## Introduction

Adenoid cystic carcinoma (ACC) is an uncommon malignant tumor that originates from the epithelial cells of the salivary glands. ACC was initially reported by Robin et al. in 1853 and later named ACC by Spies et al. in 1930 (Papaspyrou et al. 2011). This type of cancer is primarily found in the head and neck region, and it represents only 1–5% of all malignant tumors in this area (Husain et al. 2013; Amit et al. 2017; Ali et al. 2016).

Head and neck adenoid cystic carcinoma (HNACC) is typically treated with surgery followed by adjuvant radiotherapy (National Comprehensive Cancer Network. Head and Neck Cancers). Adjuvant radiotherapy is particularly effective in improving local and regional control in patients with intermediate to high risk (Chen et al. 2020; Terhaard et al. 2005). However, the role of chemotherapy in adjuvant therapy remains controversial, as only a few relevant reports are available (Samant et al. 2012; Hsieh et al. 2016). The National Comprehensive Cancer Network (NCCN) guidelines suggest that patients with HNACC and high-risk factors may consider adjuvant chemotherapy (National Comprehensive Cancer Network. Head and Neck Cancers). In contrast, the American Society of Clinical Oncology guidelines advise against routine use of concurrent chemotherapy as a standard treatment for salivary gland cancer patients receiving adjuvant radiotherapy, except in the context of clinical trials (Geiger et al. 2021). Similarly, the guidelines from the German Society of Radiation Oncology recommend against routinely adding concurrent chemotherapy for salivary gland cancer patients, as ongoing prospective randomized trials are being conducted to evaluate its effectiveness (von et al. 2022). Currently, the efficacy of adjuvant chemoradiotherapy versus adjuvant radiotherapy for the treatment of salivary gland cancers with adverse features is being investigated in the ongoing phase III randomized controlled trial RTOG-1008 (Joshi and Broughman 2021).

Despite the absence of level I evidence, some radiation oncologists have incorporated the RTOG-1008 trial protocol of including adjuvant concurrent chemotherapy in the treatment of HNACC. While waiting for the results of ongoing clinical trials, this study retrospectively analyzed HNACC patients who received adjuvant radiotherapy at a single center. The objective of the study was to assess the effectiveness and toxicity of platinum-based adjuvant chemoradiotherapy (POCRT) in comparison to postoperative radiotherapy (PORT).

## Methods and materials

### Patients

This retrospective study aimed to analyze patients diagnosed with HNACC at our center from January 2010 to April 2020. Inclusion criteria were as follows: (1) patients who underwent surgery with curative intent and completed radiotherapy at our center; (2) non-recurrent or metastatic disease; (3) patients with complete pathological reports and follow-up data; (4) absence of a multiple primary tumor; and (5) chemotherapy regimen based on platinum. The data were reviewed under an institutional review board-approved retrospective protocol.

### Treatment

In this study, all patients underwent surgery at the primary site, with some also receiving neck dissection. The surgical approach was determined by the surgeon, taking into account the patient’s medical history, clinical examination, imaging data, and intraoperative exploration. Pathological risk factors included pathological stage T3-4, N1-3, perineural invasion (PNI), lymphovascular invasion (LVI), R1-2 resections or the presence of histologic solid component. Furthermore, all patients included in this study received postoperative intensity-modulated radiotherapy (IMRT). Clinical target volume (CTV) for all patients included the tumor bed with a 1–2 cm margin and prescribed 60–66 Gy. For patients with pathological risk factors, a boost therapy was administered, with the dose determined by the responsible radiation oncologist. A subset of patients underwent skull base and neck irradiation, with the specific protocol tailored by the attending radiation oncologist based on individual circumstances. Some patients received concurrent platinum-based chemotherapy, and the choice of adjuvant therapy was ultimately determined by a multidisciplinary team discussion. The standard chemotherapy regimen was usually based on cisplatin or nedaplatin, at a dose of 80–100 mg/m^2^ q3w or 30–40 mg/m^2^ qw. Some patients received tri-weekly lobaplatin at a dose of 50 mg/m^2^ or tri-weekly oxaliplatin at a dose of 130 mg/m^2^. All patients included in this study completed the treatment.

### Follow-up strategy and end points

To evaluate treatment response and toxicity, patients were followed up weekly at the outpatient clinic during the course of radiotherapy. Acute radiation-related toxicity was assessed based on the toxicity criteria of the Radiation Therapy Oncology Group and the European Organization for Research and Treatment of Cancer (Cox 1995). Subsequent clinical follow-up was scheduled every 3 months for the first year, every 6 months for the second and third years, and then annually thereafter. The follow-up period ended on April 30, 2023, or the date of death. Suspected recurrent or metastatic lesions were biopsied to confirm disease recurrence. The primary endpoint of this study was local–regional control (LRC), defined as the time from the start of treatment to the first recurrence at the local or regional site, whichever occurred earlier. Secondary endpoints included distant metastasis-free survival (DMFS), disease-free survival (DFS), and overall survival (OS). DMFS was defined as the time from the start of treatment to the first recurrence at a distant site, or death, whichever occurred earlier. DFS was defined as the time from the start of treatment to tumor recurrence, or death, whichever occurred earlier. OS was defined as the time from the start of treatment to death.

### Statistical analysis

Patients were divided into two groups, PORT and POCRT, according to whether they received adjuvant concurrent chemotherapy. Categorical data were presented as frequencies and percentages, and were compared using chi-square tests with continuity correction or Fisher’s exact test. The outcomes between the PORT group and the POCRT group were analyzed using the nearest neighbor matching method within propensity score matching (PSM). The matched baseline data encompassed variables such as gender, age, smoking, alcohol consumption, primary site, pathological T stage, pathological N status, perineural invasion (PNI), lymphovascular invasion (LVI), histologic solid component, resection status, skull base RT and neck RT. For LRC with death as the only competing risk, the cumulative incidence function was used to estimate locoregional failure rates, and Gray’s test was used to compare groups (Gray 1988). For DMFS, DFS, and OS, the Kaplan–Meier method was used to estimate the failure rates, and the log-rank test was used to compare patient groups. Multivariate analyses were performed using Competing risks regression or Cox proportional hazards regression models, incorporating adjuvant concurrent chemotherapy, clinicopathological characteristics and skull base/neck RT. All analyses were two-sided, and the significance level was set at P < 0.05.

## Results

### Clinical characteristics and treatment details

From January 2010 to April 2020, a total of 504 patients with pathologically diagnosed HNACC were treated at our center. The following patients were excluded from this study: 155 patients who did not undergo radical surgery combined with postoperative IMRT, 82 patients with recurrence or metastasis, 56 patients with incomplete data or lost follow-up, 3 patients with concomitant other malignancies, and 2 patients who received docetaxel chemotherapy. Finally, 206 patients were included in the study, and all patients were restaged according to the 8th edition of the American Joint Committee on Cancer criteria. The entire cohort included 147 patients who received PORT (71.4%) and 59 patients who received POCRT (28.6%). Within the entire cohort, 15.5% (n = 32) of patients underwent neck dissection. All patients underwent IMRT to a median dose of 67.5 Gy (range 60–74 Gy). Patients undergoing PORT alone were treated to a median dose of 68 Gy (range 60–74 Gy), and those undergoing POCRT were treated to a median dose of 67 Gy (range 60–70 Gy). The skull base was treated in 64.6% (n = 133) of patients (PORT group = 89, 60.5%; POCRT group = 44, 74.6%), and the neck was irradiated in 62.6% (n = 129) of patients (PORT group = 95, 64.6%; POCRT group = 34, 57.6%). The POCRT group consisted of 30 patients (50.8%) who received cisplatin chemotherapy, 12 patients (20.3%) who received nedaplatin chemotherapy, 12 patients (20.3%) who received lobaplatin chemotherapy, and 5 patients (8.5%) who received oxaliplatin chemotherapy. Among the POCRT group, 69.5% (n = 41) underwent a tri-weekly regimen of chemotherapy, while the remaining 30.5% (n = 18) received weekly chemotherapy. The use of POCRT was associated with the primary location (P = 0.048*) and pathological T stage (P = 0.011*). Propensity score matching was used to match 51 pairs of patients who received PORT or POCRT, and patient characteristics were balanced across all covariates. The clinical characteristics of the two cohorts are summarized in Table 1.

**Table 1:** Clinical characteristics of PORT and POCRT groups in the whole cohort and matched cohort

Parameters	The entire cohort (n = 206)			The matched cohort (n = 102)		
	PORT (n = 147, %)	POCRT (n = 59, %)	P value	PORT (n = 51, %)	POCRT (n = 51, %)	P value
Sex			1.000			0.843
Female	78 (53.1)	32 (54.2)		25 (49.0)	27 (52.9)	
Male	69 (46.9)	27 (45.8)		26 (51.0)	24 (47.1)	
Age, y (range 15–78 y, median 43 y)			1.000			0.796
< = 60	123 (83.7)	49 (83.1)		41 (80.4)	43 (84.3)	
> 60	24 (16.3)	10 (16.9)		10 (19.6)	8 (15.7)	
Smoke			0.644			0.715
No	127 (86.4)	53 (89.8)		48 (94.1)	46 (90.2)	
Yes	20 (13.6)	6 (10.2)		3 (5.9)	5 (9.8)	
Alcohol			0.240			1.000
No	134 (91.2)	57 (96.6)		49 (96.1)	49 (96.1)	
Yes	13 (8.8)	2 (3.4)		2 (3.9)	2 (3.9)	
Primary location			0.048*			0.872
Major salivary	59 (40.1)	14 (23.7)		14 (27.5)	14 (27.5)	
Minor salivary	73 (49.7)	34 (57.6)		26 (51.0)	28 (54.9)	
Lacrimal	15 (10.2)	11 (18.6)		11 (21.6)	9 (17.6)	
pT stage			0.011*			0.484
T1-2	64 (43.5)	14 (23.7)		10 (19.6)	14 (27.5)	
T3-4	83 (56.5)	45 (76.3)		41 (80.4)	37 (72.5)	
pN positive			1.000			1.000
No	140 (95.2)	56 (94.9)		50 (98.0)	49 (96.1)	
Yes	7 (4.8)	3 (5.1)		1 (2.0)	2 (3.9)	
PNI			0.200			1.000
No	58 (39.5)	17 (28.8)		17 (33.3)	17 (33.3)	
Yes	89 (60.5)	42 (71.2)		34 (66.7)	34 (66.7)	
LVI			0.106			0.678
No	141 (95.9)	53 (89.8)		47 (92.2)	49 (96.1)	
Yes	6 (4.1)	6 (10.2)		4 (7.8)	2 (3.9)	
Histologic solid component			0.531			1.000
Absence	57 (38.8)	26 (44.1)		20 (39.2)	21 (41.2)	
Presence	90 (61.2)	33 (55.9)		31 (60.8)	30 (58.8)	
Resection status			0.098			0.625
R0	97 (66.0)	46 (78.0)		42 (82.4)	39 (76.5)	
R1-2	50 (34.0)	13 (22.0)		9 (17.6)	12 (23.5)	
Skull base RT			0.076			0.095
No	58 (39.5)	15 (25.4)		22 (43.1)	13 (25.5)	
Yes	89 (60.5)	44 (74.6)		29 (56.9)	38 (74.5)	
Neck RT			0.426			0.692
No	52 (35.4)	25 (42.4)		26 (51.0)	23 (45.1)	
Yes	95 (64.6)	34 (57.6)		25 (49.0)	28 (54.9)	

*PORT* postoperative radiotherapy, *POCRT* postoperative chemoradiotherapy, *PNI* perineural invasion, *LVI* lymphovascular invasion, *R0* complete resection, *R1-2* microscopic or macroscopic positive

^*^Statistically significant difference (P value < 0.05)

### Clinical outcomes and patterns of failure

After a median follow-up of 73.5 months (range, 15–227 months), 21 (10.2%) of the 206 patients experienced local–regional failure, 70 (34.0%) developed distant metastasis, and 47 (22.8%) died (42 from cancer, 5 from non-cancer-related diseases or accidents). The 3-, 5-, and 10-yr LRC for the cohort were 92.0%, 90.6%, and 86.9%, respectively. The 3-, 5-, and 10-yr DMFS were 76.1%, 68.5%, and 56.7%, respectively. The 3-, 5-, and 10-yr DFS were 73.2%, 65.0%, and 54.8%, respectively. The 3-, 5-, and 10-yr OS were 91.7%, 85.3%, and 67.0%, respectively.

The most frequent pattern of failure was distant metastasis, which occurred in a median time of 30 months (range, 4–116 months). The lung was the most commonly affected site of distant metastasis (80%), followed by bone (19%), liver (17%), and brain (11%). The median time to local–regional failure was 22 months (range, 1–98 months), with 90.9% of these failures occurring in high-dose areas. Table 2 provides a detailed summary of the characteristics of patients who experienced local–regional failure.

**Table 2:** Patterns of failure of the 21 patients who developed locoregional failures after adjuvant therapy

Case	Group	Primary location	pT	N stage	PNI	LVI	R1-2^#^	Local failure	Regional failure	Time to failure, month	Time to death, month
1	PORT	Lacrimal gland	T3	N0	+	–	+	In field	–	4	Alive till last visit
2	PORT	Lacrimal gland	T2	N0	+	–	+	In field	–	36	Alive till last visit
3	PORT	Nasal cavity	T4a	N0	+	–	–	In field	–	11	28
4	PORT	Nasal cavity	T4a	N0	+	–	+	In field	–	1	35
5	PORT	Nasopharynx	T3	N0	+	–	+	In field	–	51	Alive till last visit
6	PORT	Nasal cavity	T4a	N0	+	–	–	In field	–	56	81
7	PORT	Hard palate	T3	N0	+	–	–	–	In-field (Level III) Out-field (Level IV and Va)	66	84
8	PORT	Hard palate	T4a	N0	+	+	–	In field	In-field (Level Ib, II and III)	26	33
9	PORT	Maxillary sinus	T3	N0	+	–	+	In field	–	22	41
10	PORT	Lacrimal gland	T4	N0	+	+	+	In field	–	24	30
11	PORT	Parotid gland	T4a	N1 (Level II)	+	–	+	In field	In field (Level II and V)	5	32
12	PORT	Submandibular gland	T4a	N0	+	–	+	In field	–	8	Alive till last visit
13	POCRT	Lacrimal gland	T3	N0	+	+	+	In field	–	12	32
14	PORT	Soft palate	T4	N0	–	–	+	In field	–	7	Alive till last visit
15	PORT	Lacrimal gland	T4	N0	+	–	–	Out – field	–	86	Alive till last visit
16	PORT	Maxillary sinus	T4a	N0	–	–	+	In field	–	16	Alive till last visit
17	PORT	Maxillary sinus	T4a	N0	+	–	–	–	In field (Level Ib)	98	111
18	PORT	Submandibular gland	T2	N2b (Level II)	+	–	+	In field	–	11	17
19	PORT	Maxillary sinus	T4b	N1 (Level Ib)	–	–	+	In field	–	4	146
20	POCRT	Maxillary sinus	T4a	N0	–	–	+	In field	–	61	Alive till last visit
21	PORT	Parotid gland	T3	N0	+	–	+	–	In field (Level II)	28	Alive till last visit

*PORT* postoperative radiotherapy, *POCRT* postoperative chemoradiotherapy, *PNI* perineural invasion, *LVI* lymphovascular invasion, # microscopic or macroscopic positive

### Univariable and multivariable analysis

**Fig. 1:**
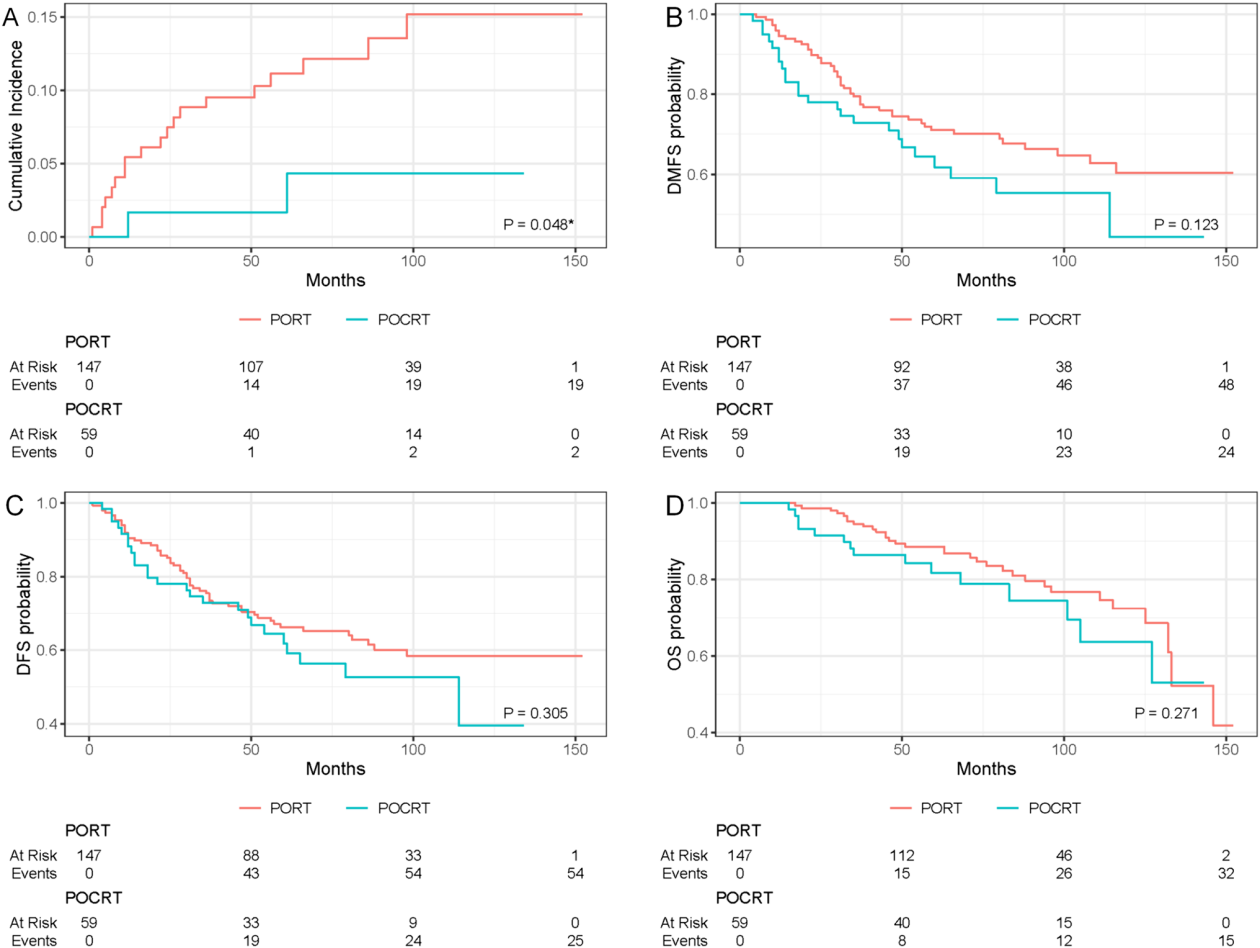
LRFCI, DMFS, DFS, and OS rates in patients with HNACC treated with PORT or POCRT before propensity score matching. *LRFCI* locoregional failure cumulative incidence, *DMFS* distant metastasis free survival, *DFS* disease free survival, *OS* overall survival, *PORT* postoperative radiotherapy, *POCRT* postoperative chemoradiotherapy. P values were calculated by Gray's test or log-rank test, *Statistically significant difference (P value < 0.05)

**Fig. 2:**
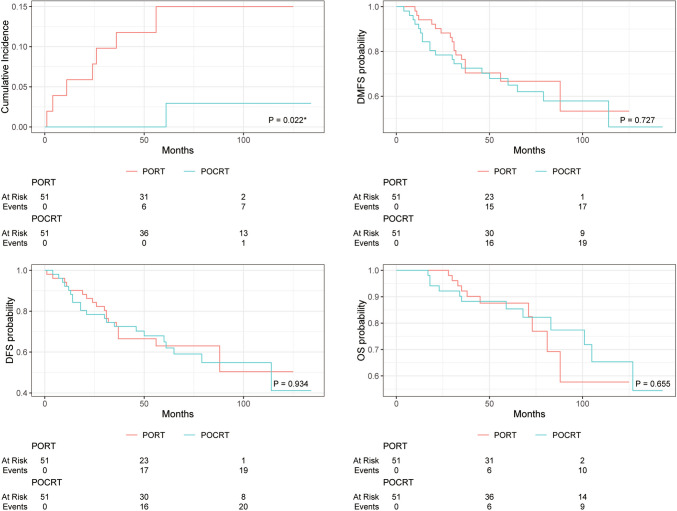
LRFCI, DMFS, DFS, and OS rates in patients with HNACC treated with PORT or POCRT after propensity score matching. Abbreviations: *LRFCI* locoregional failure cumulative incidence, *DMFS* distant metastasis free survival, *DFS* disease free survival, *OS* overall survival, *PORT* postoperative radiotherapy, *POCRT* postoperative chemoradiotherapy. P values were calculated by Gray's test or log-rank test, *Statistically significant difference (P value < 0.05)

Within the entire cohort, a comparison between the POCRT group and patients undergoing PORT indicated superior locoregional control (LRC) after accounting for competing risk events (P = 0.048*, Gray’s test, Fig. 1A). In the POCRT group, the 3-year, 5-year, and 10-year locoregional failure cumulative incidence rates were 1.7%, 1.7%, and 4.3% respectively, while in the PORT group, they were 9.5%, 11.2%, and 15.2%. However, no statistically significant differences were observed between the two groups in terms of DMFS, DFS, and OS (all P > 0.05, Log-rank test, Fig. 1B–D). To minimize inherent selection bias in the retrospective cohort, propensity score matching was used to balance the PORT and POCRT groups. Within the matched cohort, the POCRT group continued to demonstrate superior LRC compared to the PORT group (P = 0.022*, Gray's test, Fig. 2A). No significant differences were observed between the POCRT and PORT groups in terms of DMFS, DFS, and OS in the matched cohort (all P > 0.05, Log-rank test, Fig. 2B–D).

Multivariate analysis, which included adjuvant concurrent chemotherapy, clinical-pathological factors and skull base/neck RT, identified adjuvant concurrent chemotherapy as an independent prognostic factor for LRC (Competing risks regression, HR = 0.144, 95% CI 0.026–0.802, P = 0.027*, Table 3). Independent prognostic factors for LRC also included pathological T stage, pathological N status, PNI, LVI, resection status and neck RT. PNI, LVI and histologic solid component were independent prognostic factors for DMFS, and pathological N status, PNI, LVI, histologic solid component, and resection status were independent prognostic factors for DFS, and pathological N status, PNI, LVI and histologic solid component were independent prognostic factors for OS (Cox proportional hazards regression, all P < 0.05*, Table 3). Adjuvant concurrent chemotherapy was not an independent prognostic factor for DMFS, DFS, or OS.

**Table 3:** Multivariable analyses of clinicopathological factors by outcomes

Outcomes	HR (95% CI)	P value
LRC (Competing risks regression)		
Chemotherapy (POCRT vs. PORT)	0.144 (0.026–0.802)	0.027*
Primary location		
Major salivary	Ref	
Minor salivary	0.548 (0.100–2.993)	0.490
Lacrimal	0.310 (0.022–3.325)	0.310
pT stage (T3-4 vs. T1-2)	10.162 (2.103–49.114)	0.004*
pN positive (Yes vs. No)	14.582 (3.198–66.488)	< 0.001*
PNI (Yes vs. No)	3.819 (1.140–12.795)	0.030*
LVI (Yes vs. No)	4.333 (1.059–17.727)	0.041*
Histologic solid component (Presence vs. Absence)	0.391 (0.120–1.272)	0.120
Resection status (R1-2 vs. R0)	10.326 (3.074–34.687)	< 0.001*
Skull base RT (Yes vs. No)	0.712 (0.201–2.517)	0.600
Neck RT (Yes vs. No)	0.131 (0.022–0.772)	0.025*
DMFS (Cox proportional hazards regression)		
Chemotherapy (POCRT vs. PORT)	1.484 (0.873–2.524)	0.145
Primary location		
Major salivary	Ref	
Minor salivary	0.937 (0.329–2.669)	0.904
Lacrimal	0.576 (0.257–1.291)	0.180
pT stage (T3-4 vs. T1-2)	1.449 (0.850–2.469)	0.173
pN positive (Yes vs. No)	2.170 (0.954–4.936)	0.065
PNI (Yes vs. No)	2.596 (1.414–4.765)	0.002*
LVI (Yes vs. No)	3.216 (1.467–7.048)	0.004*
Histologic solid component (Presence vs. Absence)	4.602 (2.393–8.848)	< 0.001*
Resection status (R1-2 vs. R0)	1.572 (0.917–2.695)	0.100
Skull base RT (Yes vs. No)	1.557 (0.890–2.725)	0.121
Neck RT (Yes vs. No)	1.207 (0.598–2.438)	0.599
DFS (Cox proportional hazards regression)		
Chemotherapy (POCRT vs. PORT)	1.215 (0.729–2.026)	0.455
Primary location		
Major salivary	Ref	
Minor salivary	0.725 (0.274–1.918)	0.517
Lacrimal	0.543 (0.262–1.125)	0.100
pT stage (T3-4 vs. T1-2)	1.603 (0.956–2.688)	0.074
pN positive (Yes vs. No)	2.780 (1.207–6.406)	0.016*
PNI (Yes vs. No)	2.269 (1.300–3.962)	0.004*
LVI (Yes vs. No)	2.430 (1.127–5.239)	0.023*
Histologic solid component (Presence vs. Absence)	2.955 (1.680–5.200)	< 0.001*
Resection status (R1-2 vs. R0)	2.019 (1.233–3.305)	0.005*
Skull base RT (Yes vs. No)	1.321 (0.779–2.242)	0.301
Neck RT (Yes vs. No)	1.082 (0.555–2.110)	0.818
OS (Cox proportional hazards regression)		
Chemotherapy (POCRT vs. PORT)	1.279 (0.636–2.573)	0.490
Primary location		
Major salivary	Ref	
Minor salivary	1.083 (0.287–4.091)	0.906
Lacrimal	0.775 (0.283–2.120)	0.619
pT stage (T3-4 vs. T1-2)	1.197 (0.630–2.273)	0.583
pN positive (Yes vs. No)	2.471 (1.018–6.000)	0.046*
PNI (Yes vs. No)	4.512 (1.949–10.447)	< 0.001*
LVI (Yes vs. No)	5.502 (2.176–13.912)	< 0.001*
Histologic solid component (Presence vs. Absence)	4.946 (1.913–12.789)	< 0.001*
Resection status (R1-2 vs. R0)	1.283 (0.653–2.523)	0.470
Skull base RT (Yes vs. No)	1.182 (0.573–2.439)	0.651
Neck RT (Yes vs. No)	0.715 (0.296–1.728)	0.456

*LRC* locoregional control, *DMFS* distant metastasis free survival, *DFS* disease free survival, *OS* overall survival, *PORT* postoperative radiotherapy, *POCRT* postoperative chemoradiotherapy, *HR* hazard ratio, *CI* confidence interval, *PNI* perineural invasion, *LVI* lymphovascular invasion, *RT* radiotherapy

*Statistically significant difference (P value < 0.05)

### Acute toxicities

The acute toxicities during radiotherapy were evaluated and listed in Table 4, with comparison between the PORT and POCRT groups. Six patients experienced unscheduled treatment interruptions during radiotherapy, with five patients interrupting radiotherapy due to grade 4 toxicity reactions and one patient interrupting radiotherapy due to nasal bleeding. All patients resumed treatment within one week after the interruption and completed the full course of treatment. Overall, compared with the PORT group, the POCRT group had a higher incidence of unscheduled radiotherapy interruptions, but the difference was not significant (P = 0.057). In addition, the POCRT group had higher incidences of upper gastrointestinal toxicity and hematologic toxicities, including leukopenia, neutropenia, and anemia (all P < 0.05*). No grade 4 hematologic toxicity or treatment-related deaths were observed.

**Table 4:** Acute radiation-related toxicities in the 206 patients of the entire cohort

Toxicity	PORT (n = 147, %)	POCRT (n = 59, %)	P value
Unscheduled interruption			0.057
No	145 (98.6)	55 (93.2)	
Yes	2 (1.4)	4 (6.8)	
Any toxicity			0.429
G0-2	135 (91.8)	52 (88.1)	
G3-4	12 (8.2)	7 (11.9)	
Skin			0.324
G0-2	145 (98.6)	57 (96.6)	
G3-4	2 (1.4)	2 (3.4)	
Mucous membrane			1.000
G0-2	138 (93.9)	55 (93.2)	
G3-4	9 (6.1)	4 (6.8)	
Eye			0.691
G0-1	142 (96.6)	56 (94.9)	
G2-3	5 (3.4)	3 (5.1)	
Salivary gland			0.512
G0	45 (30.6)	21 (35.6)	
G1-2	102 (69.4)	38 (64.4)	
Pharynx and esophagus			0.184
G0-1	137 (93.2)	58 (98.3)	
G2-3	10 (6.8)	1 (1.7)	
Upper G.I			< 0.001*
G0	96 (65.3)	21 (35.6)	
G1	51 (34.7)	38 (64.4)	
Hematologic WBC			< 0.001*
G0-1	142 (96.6)	44 (74.6)	
G2-3	5 (3.4)	15 (25.4)	
Platelets			0.626
G0	144 (98.0)	57 (96.6)	
G1-2	3 (2.0)	2 (3.4)	
Neutrophils			< 0.001*
G0-1	143 (97.3)	44 (74.6)	
G2-3	4 (2.7)	15 (25.4)	
Hemoglobin			0.024*
G0-1	146 (99.3)	55 (93.2)	
G2-3	1 (0.7)	4 (6.8)	

*PORT* postoperative radiotherapy, *POCRT* postoperative chemoradiotherapy, *upper G.I.* upper gastrointestinal, *WBC* white blood cell

*Statistically significant difference (P value < 0.05)

## Discussion

In this single-center cohort of patients with HNACC, our study revealed that LRC of those patients treated with POCRT might be superior to those treated with PORT after accounting for competing risk events (P = 0.048* in the entire cohort, P = 0.022* in the matched cohort). However, POCRT did not show any improvement in DMFS, DFS and OS. Additionally, upper gastrointestinal and hematologic toxicities were more frequent in the POCRT group (all P < 0.05*).

The 5-yr LRC, DMFS, DFS, and OS for this study were 90.6%, 68.5%, 65.0%, and 85.3%, respectively, which were similar to the clinical outcomes reported by the French National Network on rare head and neck cancers (5-yr MFS, RFS, and OS were 62%, 64%, and 85%, respectively) in their prospective cohort (Atallah et al. 2020). Surgery remains the primary treatment for HNACC, but the complex anatomy of the head and neck often makes complete removal of high T-stage tumors difficult. Therefore, postoperative radiotherapy is an important adjuvant treatment for HNACC. The NCCN guidelines recommend adjuvant radiotherapy for all HNACC patients after surgery, and for HNACC without high-risk factors, the evidence is category 2B (National Comprehensive Cancer Network. Head and Neck Cancers). Overall, the patients included in this cohort received consistent radiotherapy techniques and achieved satisfactory local–regional control.

The addition of chemotherapy in adjuvant therapy lacks high-level evidence. However, recent data from a large national study show that the use of POCRT in HNACC has significantly increased, even in the absence of new category I evidence or clinical trial data (Gordon et al. 2023). Therefore, it is of clinical significance to clarify the role and beneficiary population of POCRT. Baseline characteristics of this study indicate that patients with minor salivary/lacrimal disease or high T-stage tumors are more likely to receive POCRT. This is likely due to the need to preserve organ function, which limits the extent of surgical resection and therefore strengthens the intensity of adjuvant radiotherapy. In the present study, LRC of HNACC treated with POCRT might be superior to those treated with PORT after accounting for competing risk events (P = 0.048* in the entire cohort, P = 0.022* in the matched cohort), and multivariate analysis showed that POCRT was an independent prognostic factor for LRC (Competing risks regression, HR = 0.144, 95% CI 0.026–0.802, P = 0.027*), suggesting that adding platinum-based adjuvant concurrent chemotherapy may be an effective method to further improve the local–regional control of HNACC. This study obtained consistent results in a relatively large sample compared with previous studies. Schoenfeld et al. included 35 patients with salivary gland cancer, and the 3-yr LRC rate in the POCRT group was 92% (Schoenfeld et al. 2012). Hsieh et al. constructed a multicenter cohort of 91 patients with salivary gland ACC, of which 33 received POCRT, and the 5-yr LRC rate in the POCRT group was 97% (Hsieh et al. 2016).

Previously conducted clinical trials have indicated that postoperative chemoradiotherapy, as opposed to postoperative radiotherapy alone, can improve DFS in head and neck squamous cell carcinoma (Bernier et al. 2004; Cooper et al. 2004). However, a similar conclusion was not reached in this study on HNACC. HNACC is a highly recurrent risk tumor, with distant metastasis and local regional recurrence being primary patterns of failure in many studies (Atallah et al. 2020; Amit et al. 2014; van et al. 2013). In the present study, although POCRT is unlikely to decrease the risk of distant metastasis in HNACC patients, it may enhance local regional control and thus improve treatment efficacy, which is particularly important given the difficulty of salvage treatment for recurrent HNACC. To improve the overall survival, effective chemotherapeutic agents for systemic treatment may be necessary.

The toxicity associated with POCRT treatment is a significant concern. Previous prospective randomized trials have demonstrated that POCRT may result in increased grade 3–4 acute toxicity compared to PORT alone (Bernier et al. 2004; Cooper et al. 2004). However, this study did not observe a significant increase in grade 3–4 acute toxicity associated with POCRT, possibly due to the large sample size difference between the two groups. Furthermore, although not statistically significant, the POCRT group showed a higher incidence of unscheduled treatment interruptions, which could negatively impact the prognosis of patients by reducing the local–regional control rate (Maciejewski et al. 1989; Suwinski et al. 2003). In this study, upper gastrointestinal and hematological toxicities were more common in the POCRT group. Based on these findings, radiation oncologists should exercise caution when applying POCRT as an adjuvant treatment for HNACC.

This study presents some limitations. Firstly, while the sample size is larger compared to previous reported studies, this research remains retrospective and the follow-up time remains relatively limited given the protracted course of HNACC. Thus, the results of this study should be interpreted with caution. Secondly, the lack of clinical pathological features, such as histological grading, may have biased the results. However, histological description seems to be operator dependent, and there is no standardized protocol at our center, it is difficult to include this variable. Thirdly, the retrospective analyses were unable to control the effects of many other potential confounders, and inconsistent chemotherapy regimens also may act as a confounding factor. Nonetheless, conducting prospective clinical studies on HNACC, a rare type of cancer, is challenging. Therefore, we suggest that this study may provide useful information for radiation oncologists, particularly regarding the potential benefits of adding chemotherapy to adjuvant treatment in HNACC patients. To summarize, platinum-based adjuvant chemoradiotherapy may be a more effective treatment modality than postoperative radiotherapy alone in reducing local–regional failure in HNACC patients in the IMRT era, and these findings require validation through further studies.

## Data Availability

All relevant data were uploaded onto the Research Data Deposit public platform (www.researchdata.org.cn) with the approval number as RDDA2023688096.
